# Durable Response in Histiocytic Sarcoma After Allogeneic Stem Cell Transplantation: A Case Report

**DOI:** 10.3390/hematolrep18010002

**Published:** 2025-12-22

**Authors:** Stefania Oliva, Jessica Gill, Elia Boccellato, Umberto Mortara, Luca Molinaro, Laura Godio, Elena Sieni, Anna Maria Buccoliero, Irene Dogliotti, Alessandro Busca, Elena Califaretti, Bruno Benedetto, Luisa Giaccone

**Affiliations:** 1 Division of Hematology, A.O.U. Città della Salute e della Scienza di Torino, 10126 Torino, Italy; 2 Department of Hematology, Santa Croce e Carle Hospital, 12100 Cuneo, Italy; 3 Department of Scienze Mediche, Anatomia Patologica 1U, A.O.U. Città della Salute e della Scienza di Torino, 10126 Torino, Italy; 4 Department of Oncologia, Ematologia e TCSE, Azienda Ospedaliero Universitaria Meyer IRCCS, 50139 Firenze, Italy; 5 Pathology Unit, Meyer Children’s Hospital IRCCS, 50139 Florence, Italy; 6 SS Trapianto Allogenico e Terapie Cellulari, SC Ematologia U Dipartimento di Oncologia, A.O.U. Città della Salute e della Scienza di Torino, 10126 Torino, Italyluisa.giaccone@unito.it (L.G.); 7 SS Trapianto Allogenico Cellule Staminali, A.O.U. Città della Salute e della Scienza di Torino, 10126 Torino, Italy; abusca@cittadellasalute.to.it; 8 SC Medicina Nucleare, A.O.U. Città della Salute e della Scienza di Torino, Presidio Molinette, 10126 Torino, Italy; ecalifaretti@cittadellasalute.to.it; 9 Department of Molecular Biotechnology and Health Sciences, University of Torino, 10124 Torino, Italy

**Keywords:** histiocytic sarcoma case report, HLH, allogeneic stem cell transplantation, PET/CT monitoring

## Abstract

**Background and Clinical Significance:** Histiocytic sarcoma (HS) is a rare and aggressive form of malignant histiocytosis, often associated with poor prognosis. The diagnosis and management of HS are challenging due to the complexity of its pathogenesis, molecular profile, and the unclear cellular origin of histiocytic neoplasms, compounded by the limited literature on treatment strategies. **Case Presentation:** We report the case of a young patient with HS localized to the lymph nodes, spleen, and liver, who also presented with hemophagocytic lymphohistiocytosis (HLH) documented on bone marrow biopsy. Initial treatment with CHOEP-21 and ICE-21 chemotherapy resulted in only a partial metabolic response, as evidenced by a Fluorodeoxyglucose-Positron Emission Tomography (FDG-PET)/CT scan. Given the aggressive nature of the disease and the presence of HLH, an allogeneic hematopoietic stem cell transplantation (HSCT) from a matched unrelated donor was performed as consolidation therapy, leading to a progressive complete response without significant toxicity. A suspected relapse at 18 months post-transplant was excluded following a mediastinal lymph node biopsy, which revealed a benign intravascular papillary endothelial hyperplasia (IPEH). Over five years post-diagnosis and more than four years after transplantation, the patient remains in complete remission with full functional recovery. **Conclusions:** This case highlights the diagnostic and molecular challenges of HS and demonstrates the curative potential of early allogeneic HSCT, even when only partial remission is initially achieved.

## 1. Introduction

Histiocytic sarcoma (HS) is a rare, aggressive, and poorly understood neoplasm, with an incidence of 0.17 per million and a median overall survival of approximately 6 months [1,2]. It is characterized by the proliferation of cells with morphological and immunophenotypic features of mature tissue macrophages/histiocytes, which is confirmed through the exclusion of other large cell tumors, such as B-cell and T-cell lymphoma, metastatic carcinoma, and melanoma, that may present with overlapping features. The clinical diagnosis—and consequently the therapeutic decision-making—is often challenging, as this condition may be classified either as a subgroup of myeloid cancers of unknown etiology or as a lymphoproliferative disorder. Clinical manifestations can be highly variable, ranging from localized to disseminated disease, often accompanied by constitutional symptoms. Furthermore, due to the rarity of the disease, there is no consensus on prognostic factors or a standardized treatment approach, and this significantly impacts the long-term outcome of the disease. Here, we report the successful and unique long-term remission of a case of HS associated with hemophagocytic lymphohistiocytosis (HLH) in a young patient treated with standard chemotherapy followed by allogeneic hematopoietic stem cell transplantation (HSCT).

## 2. Case Report

A previously healthy 31-year-old male presented with a 3-month history of night sweats and a 2-week history of fever and fatigue. His relevant medical history included immune thrombocytopenic purpura at 10 years old, treated with steroids and intravenous immunoglobulin (IVIG); notably, he had a younger sibling who died from acute myeloid leukemia.

On admission, he presented with mild hepatosplenomegaly without superficial adenopathy. Laboratory tests revealed bi-lineage cytopenia (hemoglobin 7.1 g/dL with normal mean corpuscular volume, platelets 49 × 10^9^/L), mild elevation of liver enzymes (aspartate aminotransferase 52 UI/L, alanine transaminase 45 UI/L, gamma-glutamyl transpeptidase 144 UI/L, alkaline phosphatase 159 UI/L), hypertriglyceridemia (187 mg/dL), hyperferritinemia (2897 ng/mL), low fibrinogen, and increased inflammatory markers (C-reactive protein [CRP] 82.4 mg/L, procalcitonin 2.56 ng/mL).

Acute hematologic diseases were excluded first: a peripheral blood smear ruled out thrombotic thrombocytopenic purpura, while normal hemostatic function and the absence of hemolysis features dismissed disseminated intravascular coagulation and hemolytic anemia, respectively. Deficiencies in folic acid and cobalamin, as well as hypothyroidism, were also ruled out. Subsequently, an extensive infectious workup was performed, excluding Parvovirus B19, Human Immunodeficiency Virus (HIV), hepatitis viruses (particularly HCV), Cytomegalovirus, Epstein–Barr Virus, and SARS-CoV-2 active infections. Serological testing for Leishmania, the Vidal Wright test (Salmonella typhi, Salmonella paratyphi, and Brucella), quantiferon-TB for tuberculosis, and thick blood smear analysis for Plasmodium excluded further potential causes of hepatosplenomegaly, cytopenia, and fever.

**Figure 1:**
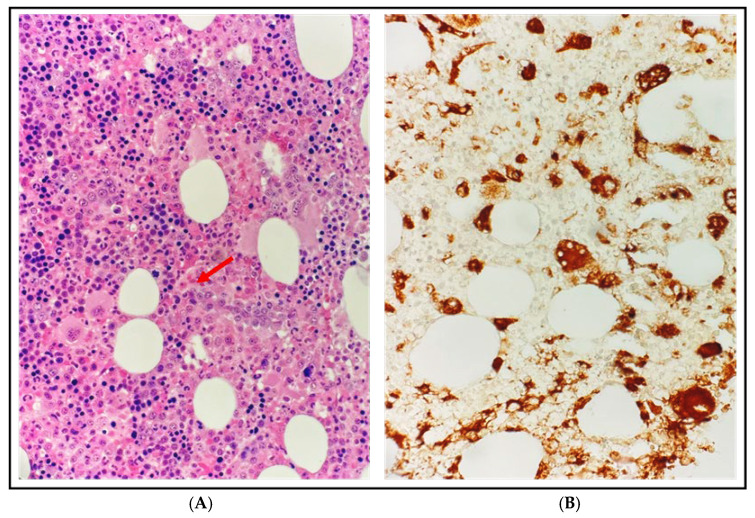
Bone marrow biopsy at diagnosis. (**A**) Dominici stain at 500× magnification: diffuse infiltration of activated histiocytes with vacuolization and phagocytosis of blood cells, particularly erythrocytes and granulocytes, consistent with hemophagocytic lymphohistiocytosis (HLH), without other proliferative disorders; the red arrow indicates an activated histiocyte engulfing 3 erythrocytes. (**B**) CD68-PGM1 immunohistochemistry at 500× magnification: histiocytes are highlighted in brown while the phagocytosed erythrocytes appear as CD68-negative elements within their cytoplasm.

**Figure 2:**
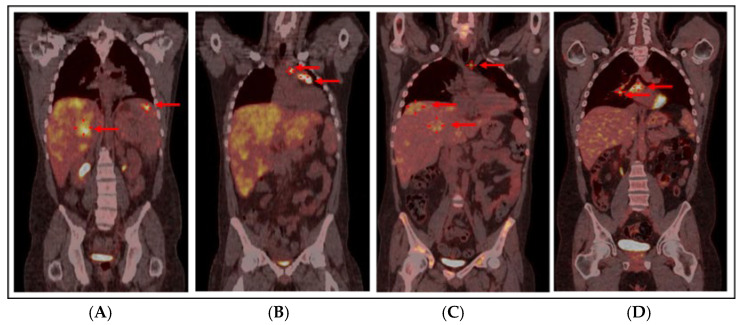
PET/CT scan at diagnosis: red arrows show extensive liver (SUV max 7.1) and splenic (SUV max 7) hypermetabolism (**A**) and lymphadenopathies in the left pre-tracheal (SUV max 8.5), pre-vascular (SUV max 8), and anterior mediastinal (SUV max 11.9) regions (**B**). (**C**) PET/CT scan after 3 cycles of ICE-21, before allo-HSCT: PMR with complete resolution of splenic hypermetabolic sites and mild reduction in mediastinal mass (32 mm, SUV max 4.6) and paratracheal and pre-vascular hypermetabolism (red arrow, SUV max 4.8), with persistent diffuse hepatic hypermetabolism (more pronounced in segments 4 and 7, as highlighted by red arrows, SUV max 4.8) in the context of hepatomegaly. (**D**) PET/CT scan 18 months after allo-HSCT: red arrows show an increase in the mediastinal mass (40 × 30 mm, SUV max 4.5) with the appearance of new hypermetabolism in the right paratracheal (SUV max 6.5), pre-carinal (SUV max 5.4), and sub-carinal (SUV max 7.2) regions and right hilar pulmonary (SUV max 6) lymphadenopathy, consistent with IPEH. Abbreviations: PET/CT: Positron Emission Tomography/Computerized Tomography, SUV: Standardized Uptake Value, ICE-21: Ifosfamide–Carboplatin–Etoposide, allo-HSCT: Allogeneic Hematopoietic Stem Cell Transplantation, PMR: Partial Metabolic Response, IPEH: Intravascular Papillary Endothelial Hyperplasia.

With these findings, HLH was suspected: the patient met 5/8 HLH-2004 criteria with a HScore of 193 [3] and a bone marrow biopsy confirmed hemophagocytosis without evidence of lymphoma or leukemia (Figure 1). A total body computed tomography (CT) scan showed a mediastinal mass (46 × 40 mm), confirmed splenomegaly (17.5 cm) with nodular lesions, and hepatomegaly. A Fluorodeoxyglucose-Positron Emission Tomography (FDG-PET)/CT scan revealed hypermetabolic activity in the mediastinal mass (Standardized Uptake Value, SUV max 11.9) with associated hypermetabolic lymphadenopathies and extensive liver and splenic involvement (SUV max 7.1) (Figure 2A,B).

**Figure 3:**
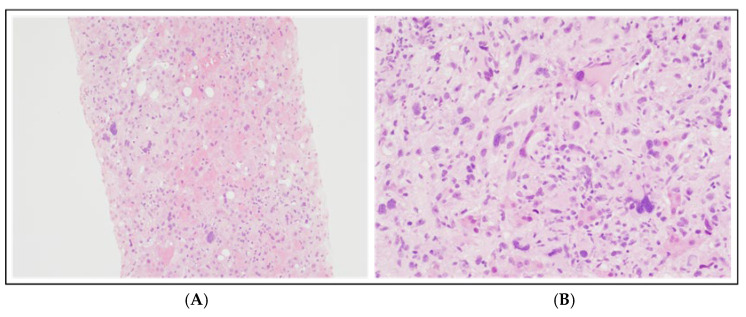
Liver biopsy at diagnosis: hematoxylin and eosin staining at 100× magnification (**A**) and 200× magnification (**B**). These histological images illustrate atypical cells with weakly eosinophilic cytoplasm and heterogeneous nuclei in shape and size, ranging from medium-sized oval or spindle-shaped elements to cells with very large and pleomorphic nuclei, and some multinucleated cells, with vesicular chromatin and prominent nucleoli.

Given the difficulty of biopsying mediastinal mass or other deep lymphadenopathies, a trans-jugular biopsy of PET-positive liver lesions was performed, showing large atypical histiocytic cells interspersed among hepatocytes and inflammatory cells (Figure 3). Immunohistochemical (IHC) studies demonstrated the expression of CD68PGM1, a key histiocytic marker, and lysozyme; CD45, CD45RO, and CD4 were also observed, while S100 immunoreactivity was variable; markers for Langerhans cells (CD207), follicular dendritic cells (CD21, CD35), myeloid cells (myeloperoxidase, CD13), and ALK were negative as well as melanocytic markers, epithelial markers, and specific mature B-/T-cell markers, with a MIB-1 of approximately 20%. Based on these findings, a diagnosis of HS was made, according to WHO 2017 criteria [4].

Histological review by two independent institutions (Brescia and Firenze) confirmed the diagnosis, showing CD163 (histiocytic marker) positivity and CD1a (Langerhans cell marker) negativity.

Molecular analysis revealed strong PD-L1 expression (80%); unfortunately MEK expression was not performed due to insufficient sample material. Nevertheless, we were able to assess a BRAF codon 600 mutation analysis after DNA extraction on stored material [Kit Maxwell-16 Tissue DNA, Promega, Cat. AS1030, Milano, Italy] followed by Real-Time PCR analysis [kit EasyPGX ready BRAF CE-IVD; Diatech Pharmacogenetics-Easy PGX qPCR instrument 96–Diatech Pharmacogenetics, Ancona, Italy], with no evidence of mutated variants.

In view of allogeneic HSCT, direct Sanger sequencing ruled out familial HLH by analyzing *UNC13-D*, *STXBP2*, *STX11*, *Rab27A*, *SH2D1A*, and *Birc-4* genes [3] on peripheral blood DNA at the Histiocytosis Laboratory in Florence, resulting in the absence of nucleotide variants with pathogenic significance in the analyzed gene portions. Concurrently, an early search for an unrelated donor was initiated, including Human Leukocyte Antigen (HLA) typing of parents to ensure a readily available backup donor.

The patient started chemotherapy in 2020 with CHOEP-21 (Cyclophosphamide 750 mg/m^2^, Doxorubicin 50 mg/m^2^, Etoposide 100 mg/m^2^, and Vincristine 1.4 mg/m^2^) combined with high-dose dexamethasone and IVIG for HLH.

Initially, there was rapid deterioration of HLH symptoms (fever, severe cytopenia, elevated ferritin, triglycerides, and CRP). After a mild improvement in blood counts, a second cycle of CHOEP-21 was administered. Restaging with PET/CT after two cycles showed partial metabolic response (PMR), with resolution of splenic and vertebral lesions, reduced hypermetabolism in paratracheal and pre-vascular lymph nodes, and reduced mediastinal mass (3.5 cm vs. 5 cm, with SUV max 4.8 vs. 11.9). However, there was persistent diffuse hyperfixation of FDG in the liver in the context of diffuse hepatomegaly.

Treatment intensification with three cycles of ICE (Ifosfamide 5g/m^2^, Carboplatin 800 mg, Etoposide 100 mg/m^2^) further improved the PMR on PET/CT (Figure 2C), showing a mild reduction in mediastinal mass (32 mm vs. 35 mm) with SUV max 4.6 (vs. 4.8), mild reduction in paratracheal (SUV max 4.8) and pre-vascular lymphadenopathy (SUV max 3.3), and hepatomegaly with diffuse hyperfixation, particularly in segments S7 (SUV max 4) and S8 (SUV max 4.4) and S4 (SUV max 3.9); splenic nodular lesions disappeared. Finally, to assess residual hepatic hyperfixation, a liver biopsy was performed, showing inflammation only but no HS or HLH; liver magnetic resonance imaging with hepato-specific contrast suggested hemosiderosis, with negative genetic testing for HFE mutations; mediastinal biopsy revealed fibrosis and fat necrosis; and bone marrow biopsy showed trilinear hyperplasia, dyserythropoiesis, and an excess of hemosiderin.

Therefore, we decided to consolidate the partial response in this aggressive disease with an allogeneic HSCT, using two additional CHOEP-21 cycles as a bridge to transplant. On May 13, 2021, with an HCT-CI score [5] of 3 due to pulmonary impairment, he underwent a myeloablative allo-HSCT from a matched unrelated female donor (10/10). The conditioning regimen was with cyclophosphamide (CY, 60 mg/kg × 2 days) and total body irradiation (TBI, 12 Gray); he received cryopreserved peripheral blood stem cells (13.52 × 10^6^ CD34+/kg) due to the pandemic; and Graft-versus-Host Disease (GVHD) prophylaxis included standard anti-thymocyte globulin (ATG, methotrexate, and cyclosporine–CsA). During the aplastic phase, the patient developed pneumonia with fever and desaturation, without microbiological findings (bronchoalveolar lavage negative). He required low-flow oxygen, broad-spectrum antibiotics, and antifungal therapy. Additional complications included grade IV mucositis and syndrome of inappropriate antidiuretic hormone secretion (SIADH), treated with intravenous sodium supplementation. Neutrophil engraftment was documented on day +16, and platelet engraftment followed on day +18. Bone marrow assessments on day +28 showed no blasts, no signs of HLH or HS, and full donor chimerism.

One month after HSCT, the patient presented with grade 2 acute GVHD with upper gastrointestinal involvement [6], successfully treated with methylprednisolone which was discontinued after 3 months. CsA was discontinued after 7 months from HSCT, without development of chronic GVHD.

The PET/CT scan at 2 months post-HSCT revealed residual mediastinal (33 mm, SUV max 4.8) and hepatic (SUV max 5) uptake, which progressively resolved; at 1 year after HSCT, only a calcified mediastinal residue of 27 × 26 mm was detectable, with an improvement entirely dependent on the graft-versus-tumor effect as no further treatment was administered.

**Figure 4:**
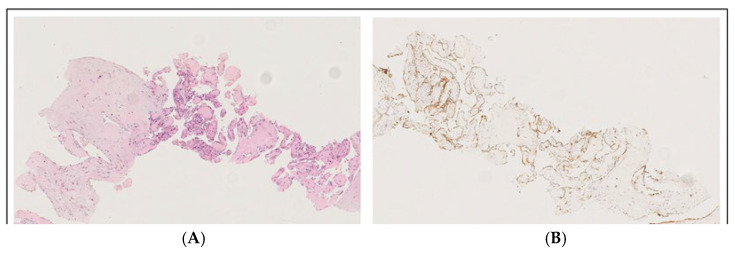
Masson’s tumor/intravascular papillary endothelial hyperplasia (IPEH): the image with hematoxylin–eosin staining at 150× magnification (**A**) shows a fibrous tissue biopsy, including a dilated vessel containing a papillary proliferation of plump endothelial cells without atypia, while anti-CD31 immunohistochemistry at 100× magnification (**B**) shows the expression of CD31, demonstrating the vascular origin.

At 18 months after HSCT, a PET/CT scan (Figure 2D) revealed an increase in the mediastinal mass (40 × 30 mm, SUV max 4.5) with new hypermetabolic mediastinal lymphadenopathies (SUV max 7.2) suspected for HS relapse. A prompt biopsy of the mediastinal mass diagnosed an intravascular papillary endothelial hyperplasia (IPEH, Figure 4), a benign vascular proliferation [7]. Given the absence of symptoms, radiological monitoring was adopted: a PET/CT scan 3 months later showed resolution of nodal uptake and stable mediastinal mass at 4 cm, confirmed at subsequent scans at 6 months and later annually.

**Figure 5:**
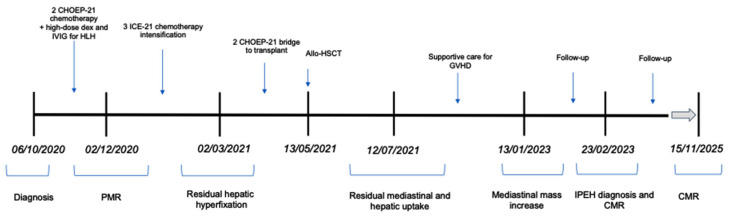
Timeline of clinical findings. Abbreviations: CHOEP-21: cyclophosphamide, doxorubicin, etoposide, vincristine; IVIG: intravenous immunoglobulin; HLH: hemophagocytic lymphohistiocytosis; ICE: ifosfamide–carboplatin–etoposide; allo-HSCT: allogeneic hematopoietic stem cell transplantation; GVHD: graft-versus-host disease; PMR: partial metabolic response; IPEH: intravascular papillary endothelial hyperplasia; CMR: complete metabolic response.

A timeline to summarize the most important clinical findings and response to therapy is shown on Figure 5.

After more than 5 years since diagnosis and more than 4 years since HSCT, the patient is in excellent general health, in remission, without signs of GVHD, and has resumed his professional and athletic activities.

## 3. Discussion

We report the clinical case of a young patient diagnosed with HS complicated by HLH who was successfully treated with chemotherapy followed by HSCT. HS is a rare, aggressive, and poorly understood hematologic malignancy. Its diagnosis and management remain particularly challenging due to its rarity, the lack of standardized treatment protocols, and the frequent difficulty in distinguishing it from other neoplasms.

A major obstacle in the diagnostic and consequently therapeutic approach to HS lies in accurate morphological and, most importantly, immunohistochemical (IHC) assessment. This requires not only the judicious use of tumor-specific markers but also a high level of diagnostic expertise. Confirming histiocytic lineage and ruling out other poorly differentiated large cell malignancies typically necessitate a broad immunophenotypic panel and, in some cases, electron microscopy. HS is characterized by the expression of various markers, including CD163, CD68, and lysozyme. CD31, CD45, CD45RO, and CD4 are also frequently expressed, while S100 staining is variably positive. In contrast, markers for Langerhans cells (CD1a, CD207), follicular dendritic cells (CD21, CD35), myeloid cells (myeloperoxidase, CD13), and ALK, as well as melanocytic, epithelial, and lineage-specific B- and T-cell markers, are typically negative. Notably, advancements in immunohistochemistry have revealed that many previously reported cases of HS were, in fact, misdiagnosed high-grade non-Hodgkin lymphomas.

In our case, the diagnosis of HS was confirmed by identifying distinctive morphological features: large, round to oval tumor cells displaying a relatively non-cohesive growth pattern and abundant eosinophilic cytoplasm, supported by a characteristic IHC profile. This was further supported by the expression of relevant immunohistochemical markers, such as CD68, PGM1, and CD45, along with the absence of other lineage-specific markers [1].

The molecular landscape of HS remains incompletely understood. However, previous studies have identified activating mutations in key oncogenic pathways, particularly PI3K/AKT/mTOR and RAS/MAPK, which appear to play a pivotal role in HS pathogenesis [8]. Notably, Egan et al. [9] reported a high frequency of genetic alterations in the RAS/MAPK pathway across all 21 HS cases analyzed, with mutations observed in NF1, MAP2K1, and PTPN11.

Emerging data suggest that targeted therapies may hold significant promise in this setting. Case reports have demonstrated that MEK inhibitors such as trametinib and BRAF inhibitors such as vemurafenib can be effective in HS patients harboring MAPK/ERK pathway mutations [10], including BRAF V600E [11], KRAS [12], and MAP2K1 [13,14]. These findings have been further corroborated by Next-Generation Sequencing (NGS) analyses of tumor tissues, which have increasingly identified BRAF V600E and other MAPK-related mutations. Moreover, kinase fusions (e.g., involving BRAF, ALK, and NTRK1) have emerged as novel therapeutic targets in histiocytic neoplasms [15].

In parallel, the role of immune checkpoint blockade has gained attention. Recent reports have highlighted overexpression of PD-L1/PD-1 in a subset of HS patients, with some achieving favorable responses to checkpoint inhibitors (CPI) [16,17]. This therapeutic rationale extends to HLH, where immune dysregulation may also be mitigated by CPI, either as part of the hemophagocytic mechanism itself [18] or in patients with overlapping HLH and PD-L1-positive HS [19].

MEK and PD-1 inhibitors have so far demonstrated favorable responses, with reported cases of responses lasting up to 30 months [12,17]. Based on these promising insights, we pursued molecular characterization in our patient, specifically assessing PD-L1 expression and BRAF V600E mutation. Despite the inherent limitations related to local diagnostic resources and the limited tumor tissue available, our analysis revealed strong PD-L1 expression, although no actionable mutations were identified. Should the patient experience future disease relapse, we plan to expand molecular profiling through advanced techniques such as NGS or whole-exome sequencing (WES) to uncover potential pathogenic drivers and inform targeted therapeutic strategies. The PD-L1 findings may also support the future use of checkpoint inhibition in this patient’s treatment course.

While these advancements offer hope, conventional chemotherapy remains the most frequently reported first-line therapy for HS. In our case, we opted for a CHOEP regimen to leverage the role of etoposide, considered the cornerstone of HLH treatment [3,20]. Following disease progression, we administered ICE as second-line therapy, in line with published reports [1,21]. Other salvage strategies include immunomodulatory agents such as thalidomide [22], also evaluated as maintenance therapy [23], and high-intensity “acute myelode-like” regimens such as CLAG-M (cladribine, cytarabine, filgrastim, mitoxantrone), although the latter is associated with considerable toxicity [21]. It is well established that responses to chemotherapy in HS are generally limited to partial remissions [20,21,22,23] and tend to be short-lived, unless consolidation with stem cell transplantation is performed. The longest chemotherapy-only remission reported [24] lasted 14 months. In this context, FDG-PET imaging, with its whole-body coverage and favorable signal-to-background ratio, may enhance the accuracy of staging and response assessment [25,26].

Among available curative strategies, HSCT has shown the most promise. Allogeneic HSCT offers the best chance for long-term survival in aggressive HS, with Zeidan et al. reporting survival of up to 12 months [20] and with some cases showing conversion from PR to CR due to the graft-versus-tumor effect [21].

While there is no standardized transplant approach, myeloablative conditioning—ranging from busulfan–cyclophosphamide [20,22] to cyclophosphamide–TBI [21]—is generally preferred when clinically feasible. Timely donor identification and early HSCT, ideally during at least a PMR, are crucial for optimizing outcomes. In cases lacking a matched donor, haploidentical HSCT may be a viable alternative, particularly with post-transplant cyclophosphamide (PTCY) prophylaxis [20]. Autologous HSCT remains a consideration for patients with comorbidities precluding allogeneic transplantation, provided there is limited or no bone marrow involvement [24,27].

In conclusion, we report one of the longest documented remissions of HS achieved through combined chemotherapy and allogeneic HSCT. This case highlights several key strengths:

− Timely diagnosis through comprehensive histopathologic and IHC evaluation.
− Integration of molecular insights, despite technical and logistical limitations.
− Strategic treatment sequencing, incorporating both HLH-targeted and HS-directed therapies.
− Successful application of curative-intent allogeneic HSCT.

We believe that the clinical and practical implications of this case may provide valuable guidance for clinicians managing these rare and complex disorders. It may assist in selecting the most appropriate chemotherapy regimen, in radiologic and histologic monitoring, in choosing the conditioning regimen for allogeneic transplantation, and in conducting comprehensive genetic and molecular evaluations to inform potential future targeted therapies.

While our work was limited by restricted molecular testing capabilities and a lack of access to broader targeted therapies, these gaps underscore the urgent need for international collaboration, access to advanced diagnostics, and clinical trials tailored to ultra-rare hematologic malignancies such as HS. Ongoing advances in molecular characterization and immunotherapy are likely to further expand the therapeutic arsenal and improve outcomes for patients with HS in the near future.

## Data Availability

No new data were created or analyzed in this study. Data sharing is not applicable to this article.
